# Efficacy of Ketorolac Tromethamine Diluted Saline Irrigant on Postoperative Sequelae in Patients Undergoing Lower Third Molar Impaction Surgery: A Prospective Clinical Study

**DOI:** 10.7759/cureus.66179

**Published:** 2024-08-05

**Authors:** Soorumsetty Ruthvik, Melvin George, Murugesan Krishnan, Santhosh P Kumar

**Affiliations:** 1 Oral and Maxillofacial Surgery, Saveetha Dental College and Hospitals, Saveetha Institute of Medical and Technical Sciences, Saveetha University, Chennai, IND

**Keywords:** ketorolac tromethamine, vas score, pain, plain saline, irrigation, surgery, lower third molar

## Abstract

Background

Lower third molar surgery is very commonly performed for minor oral surgery by an oral and maxillofacial surgeon. One of the main chief complaints that patients report back to the clinic after getting their lower third molar impaction surgery is immediate postoperative pain. In our study, we have compared the efficacy of ketorolac tromethamine diluted saline solution over plain saline solution used as an irrigant in reducing postoperative swelling and pain.

Aim

The aim of the current study is to analyse the efficiency of ketorolac tromethamine diluted saline solution over normal saline without any drug dilution in reducing postoperative sequelae like pain and swelling after surgical removal of the lower third molar.

Materials and methods

This study was carried out at the Department of Oral and Maxillofacial Surgery, Saveetha Dental College and Hospital, Saveetha Institute of Medical and Technical Sciences (SIMATS), Saveetha University, from April 2023 to July 2023. The study included 50 individuals who wanted to prophylactically get the lower third molar removed surgically. These participants were divided into two groups. One group received ketorolac diluted saline irrigant while the other group received plain saline (0.9% NaCl) as irrigant. Postoperatively, pain and swelling were evaluated in both groups. Both pain and swelling were measured preoperatively, postoperatively after 48 hours, and postoperatively after seven days. The swelling was measured using a 4-point measuring scale, and pain measurement was done using a 10-point visual analogue scale. Statistical analysis was done using Statistical Package for the Social Sciences (IBM SPSS Statistics for Windows, IBM Corp., Version 23.0, Armonk, NY). For the comparison of continuous variables between the two groups, an unpaired t-test was used. The normality of the results obtained was checked using the Shapiro-Wilk test. The results were considered statistically significant if the P-value was less than 0.05.

Results

Based on the results obtained it was found that participants who were included in the ketorolac saline group had comparatively lower postoperative pain scores than participants in the plain saline group and this was statistically significant (P=0.001). Postoperative swelling was also comparatively lower in the ketorolac tromethamine saline group but the results were not statistically significant at the end of day 7 (P=0.09).

Conclusion

Upon observing the cumulative results obtained, we conclude that ketorolac saline (10mg/100mL) was more efficacious in terms of pain reduction than the regular saline solution in impacted lower third molar surgery.

## Introduction

It is not astonishing to know that extraction of teeth was started back in 10,000-50000 BC [1]. Most of the modern instruments that we use today have evolved from the primitive designs that were actually put forward by the barbers of Europe in the late 1800s [2]. Only after the 18th century surgical removal of impacted third molars gained popularity. As we see it now, surgical removal is being done at ease with surgical handpieces but back then it was all done with the help of chisel and mallet [3]. It is because of this that people started to have a fear of visiting the dentist and this fear still continues, not because of the intraoperative procedure but because of the postoperative pain which haunts them [4].

In early Britain surgical removal of impacted teeth was considered one of the most painful and traumatic procedures, causing both physical and psychological damage to the patients [5]. Decades had passed by but the fear of getting the impacted molar removed is still persisting [6]. Lower third molar removal is a very common but challenging procedure done as part of minor oral surgery. Even though it is a very common and regularly performed procedure by oral surgeons, many concepts related to this surgery are still unclear leading to unpleasant postoperative sequelae and one of the most common and highly feared postoperative sequelae by patients is the postoperative pain and others being the swelling and trismus [7].

In the existing literature, there are several studies that tell how to control or manage these postoperative sequelae but there aren’t many studies that concentrate on novel techniques to prevent these complications [8]. Currently, postoperative pain is being managed by prescribing analgesics to the patients either postoperatively or as preemptive analgesics. Non-steroidal anti-inflammatory drugs produce analgesic as well as anti-inflammatory actions [9]. They act by inhibiting cyclooxygenase (COX) enzymes. These COX are the enzymes that help in the conversion of arachidonic acid into prostacyclins, prostaglandins and thromboxane. By inhibiting this action there is a decrease in fever, pain and inflammation [10].

Ketorolac tromethamine is one such non-steroidal anti-inflammatory drug that is FDA-approved and used for the treatment of severe to moderate kind of pain [11]. Ketorolac acts by inhibiting both COX 1 and 2. It is believed to be having higher demonstrated potency than most of the existing non-steroidal anti-inflammatory drugs. It has been proved that there is good topical action of ketorolac tromethamine also. Considering this fact we have used Ketorol DT 10mg tablet in 100mL of plain saline at room temperature as an irrigant and evaluated its potency in reduction of postoperative pain and swelling [12].

The aim of this study was to compare the efficacy of ketorolac tromethamine 10mg in 100mL saline over plain saline solution in the reduction of postoperative pain and swelling after mandibular third molar impaction surgery.

## Materials and methods

Design of the study

This was an in vivo study that was conducted in the Department of Maxillofacial Surgery, Saveetha Dental College and Hospital, Saveetha Institute of Medical Technological Sciences, Chennai. This study was conducted in the time frame of four months, from April 2023 to July 2023. Institutional Ethical Committee clearance was obtained before we began the study (IHEC/SDC/OMFS-2106/23/109). Based on the G* power calculation (Heinrich-Heine-Universität Düsseldorf, Düsseldorf, Germany) and 95% confidence interval, the total sample size was calculated to be 23 per group, and considering dropouts from the study and loss of follow up the total sample size was determined to be 50 with 25 participants in each group. After obtaining informed consent from the patients, they were allocated into respective groups by a simple random allocation technique. Allocation was followed by using a non-transparent envelope. Surgical technique was done by the same principal surgeon and assessment was carried out by the same principal investigator in both groups. Double blinding was followed to prevent bias. Both the patient and the principal investigator were not aware of the irrigant the patient received during the surgery.

Inclusion criteria

All patients requiring lower third molar impaction surgery irrespective of sex, with an age ranging from 20 to 45 years, were included.

Exclusion criteria

Patients with existing systemic illnesses such as gastrointestinal ulcers, chronic kidney disease, gastro-oesophageal reflux disorder (GERD), Crohn’s disease, and existing infection in the lower third molar were excluded.

Surgical procedure

**Figure 1:**
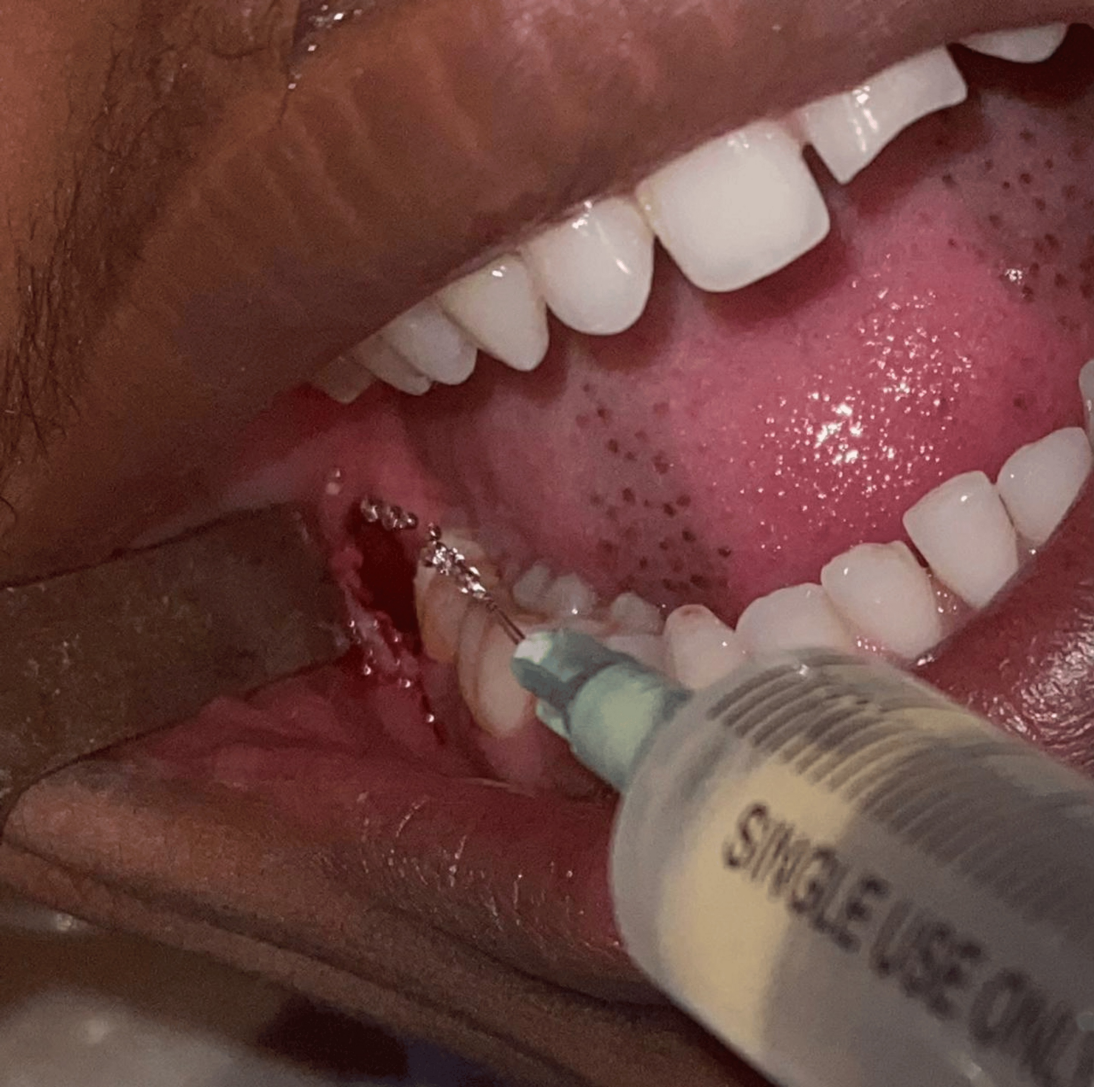
Irrigation with ketorolac tromethamine saline solution

After proper clinical and radiographic examination and diagnosis, under sterile aseptic conditions, standard scrubbing and draping were performed, and the intraoral site was irrigated with povidone-iodine solution. Lignocaine 2% with 1:80,000 adrenaline was administered as an inferior alveolar nerve block on the side planned for third molar removal. A modified Ward’s incision was placed, and a full-thickness mucoperiosteal flap was elevated, followed by bone guttering using the Moore and Gillbe technique. In group 1, patients received ketorolac tromethamine 10mg diluted in saline as an irrigant, as shown in Figure 1, while group 2 patients received plain normal saline as an irrigant. Double blinding was maintained in this study, where both the patient and the operator were unaware of the irrigant used. The irrigant was pre-loaded in opaque syringes and given to the operator by a dental assistant who was unaware of the study and which irrigant was in each syringe. The tooth was elevated and extracted, followed by bleeding control achieved through firm finger pressure with the socket packed with gauze. Suturing was performed with 3-0 silk, and postoperative medications were provided according to the protocol followed at our institution.

Follow-up

The parameters that were assessed postoperatively were pain and swelling. The swelling was assessed preoperatively, fourth day postoperatively and seventh day postoperatively. Facial measurements were taken using four stable landmark points including the corner of the mouth mid of the tragus and corner of the eye to gonion were measured by a single principle investigator. Pain assessment was done using a 10-point visual analogue scale at follow-up intervals of postoperative second day, fourth day and seventh day.

Statistical analysis

The data which was obtained was properly analysed with means and standard deviations and with a confidence interval of 95%. For statistical analysis, Statistical Package for the Social Sciences (IBM SPSS Statistics for Windows, IBM Corp., Version 23.0, Armonk, NY) was used. For a comparison of continuous variables between the two groups, an unpaired t-test was used. The normality of the results obtained was checked using the Shapiro-Wilk test. The results were considered statistically significant if the P-value was less than 0.05.

## Results

The total number of participants included in the study was 50, of which 29 (58%) were males and 21 (42%) were females, with a mean age of 25 ± 5.6 years. We divided the participants into two groups, Group 1 (N=25), in whom 10mg ketorolac tromethamine diluted saline was used as an irrigant and Group 2 (N=25) in whom plain saline was used as an irrigant. Pain and swelling were measured in all the patients at predetermined follow-up intervals.

Pain measurement

Pain was measured using a 10-point visual analogue scale which was recorded at postoperative intervals on the second day, fourth day and seventh day. The values obtained are depicted in Table 1.

**Table 1:** Comparison of pain among both the study groups Group 1: ketorolac tromethamine 10mg saline group; Group 2: plain saline group * indicates statistically significant; independent sample t-test.

Visual analogue scale	Groups	N	Mean	SD	P-value
Day 2	Group 1	25	4.12	0.81	0.001*
	Group 2	25	5.0	1.19	
Day 4	Group 1	25	2.57	1.06	0.001*
	Group 2	25	3.32	0.97	
Day 7	Group 1	25	1.27	0.88	0.001*
	Group 2	25	2.22	0.706	

Based on the results obtained, it was found that participants who used ketorolac tromethamine 10 mg diluted in saline as an irrigant reported statistically significantly lower pain levels than participants using plain saline as an irrigant on the second day (P=0.001), the fourth day (P=0.001), and the seventh day (P=0.001).

Measurement of swelling

Measurements of the face as described above were measured preoperatively and then postoperatively on the fourth day and seventh day. Four-point scale is used for the assessment of swelling. Two measurements from above mentioned stable landmarks were taken and the mean of the measurements was considered for the final assessment. The values that were obtained in mm (millimetres) are depicted in Table 2.

**Table 2:** Comparison of swelling among the study groups Group 1: ketorolac tromethamine 10mg saline group; Group 2: plain saline group; NS: non-significant * indicates statistical significant.

Swelling	Groups	N	Mean	SD	P-value
Preoperatively	Group 1	25	9.78	0.318	0.24
	Group 2	25	9.72	0.375	NS
Day 4	Group 1	25	10.16	0.308	0.001*
	Group 2	25	10.7	0.305	
Day 7	Group 1	25	9.87	0.29	0.09
	Group 2	25	9.92	0.35	NS

Based on the results that were obtained, there was a reduction in the swelling seen in the group who received 10mg ketorolac tromethamine diluted saline as irrigant compared to the normal saline irrigant group. The results obtained were statistically significant on day 4 (P=0.001) while it was not statistically significant on day 7 (P=0.09).

## Discussion

Impaction is the most commonly performed minor oral surgical procedure in the field of oral and maxillofacial surgeons. Being a very versatile procedure, it also brings in an equal amount of postoperative complications [13]. There are a lot of studies in the existing literature that focus mainly on how to reduce postoperative complications or how to manage the postoperative complications that have already occurred, but there are no studies that give a defensive solution to prevent these postoperative sequelae [14]. Most of the postoperative sequelae that are encountered can be managed by careful intraoperative modifications to traditionally followed techniques [15].

Intraoperatively to prevent the heat that is produced while guttering the bone plain saline is used in regular clinical practice. Normal saline acts as a coolant and dissipates the heat that is produced while guttering and prevents regional burn caused while performing the procedure. Plain saline usage has minimized the damage that is caused but it couldn’t prevent the postoperative sequelae that are seen [16]. Postoperative pain and swelling are two main important sequelae [17].

In our study, we used an analgesic-infused saline for irrigation. Ketorolac is a non-steroidal anti-inflammatory drug that is FDA-approved and used for the treatment of severe to moderate kind of pain. Ketorolac acts by inhibiting both COX 1 and 2 [18]. It is believed to be having higher demonstrated potency than most of the existing non-steroidal anti-inflammatory drugs. We have used commercially available ketorolac tromethamine 10mg and added it to 100mL saline solution and used it as an irrigant. The topical action of this drug has already been proven and is being used as creams and gels for maxillofacial and orthopaedic reasons. It is believed that ketorolac acts on the exposed nerve fibres in the surgical site when topically used and prevents pain production by locally inhibiting the COX pathways [18].

In this study, we have obtained a reduction of postoperative pain scores which was statistically significant in the group that received ketorolac tromethamine 10mg diluted saline as an irrigant when compared to the normal saline group. The pain relief was very spontaneous and all the patients in this group reported very low visual analogue scale scores on the immediate postoperative second day and also the seventh day. There was a significant reduction of swelling in the group that used ketorolac tromethamine diluted saline as an irrigant on immediate postoperative day 2 but in immediate postoperative day 7, there was no statistically significant difference between both the groups.

Limitations of the study

The study proved that pain and swelling reduction postoperatively can be obtained using ketorolac tromethamine diluted saline as an irrigant; the major drawback is its smaller sample size. Further studies with larger sample sizes can be done to properly analyze the efficacy of ketorolac tromethamine 10mg diluted saline as a potential irrigant. Being a single institutional study, there may be a lack of generalisability.

## Conclusions

From the data acquired and results obtained from this study, it is proven that ketorolac tromethamine 10mg diluted saline is a potential irrigant that has reduced postoperative pain and swelling in patients who underwent lower third molar impaction surgery. Since ketorolac tromethamine is very economical and available very easily, it can be employed in routine clinical practice by oral and maxillofacial surgeons. However, the efficacy of ketorolac tromethamine as a potential irrigant can be demonstrated by conducting more randomised controlled trials with a larger population.
